# Corrigendum: Characterizing a stable five-species microbial community for use in experimental evolution and ecology

**DOI:** 10.1099/mic.0.001528

**Published:** 2025-02-03

**Authors:** Meaghan Castledine, Joseph Pennycook, Arthur Newbury, Luke Lear, Zoltan Erdos, Rai Lewis, Suzanne Kay, Dirk Sanders, David Sünderhauf, Angus Buckling, Elze Hesse, Daniel Padfield

**Affiliations:** 1 Centre for Ecology and Conservation, College of Life and Environmental Sciences, University of Exeter, Penryn, Cornwall, TR10 9FE, UK; 2 University College Cork, Cork, T12 K8AF, Ireland

In the published version of the article, there was an error in the Data Availability section and in Table 1, as the reference genomes of *Stenotrophomonas* and *Variovorax* were submitted wrongly to the NCBI. The correct genomes for each strain have now been submitted, and accessions were updated accordingly.

The second sentence of the Data Availability section should read “The assembled genomes have been submitted to NCBI and are available under the following accessions: *Achromobacter veterisilvae* AB1 (CP148753.1), *Ochrobactrum teleogrylli* AB1 (JBBHKQ000000000.1), *Pseudomonas fluorescens* AB1 (CP148752), *Stenotrophomonas* sp. AB1 (2024)(JBBHKO000000000.2), and *Variovorax* sp. AB1 (2024) (JBBHKP000000000.2)”.

Table 1 should be as follows:

**Table 1.** Genome characteristics and taxonomic assignment of the five species genomes

Isolate	NCBI submission name	Sanger sequence assignment	GTDBtk assignment	16S copy no.	Genome size (bp)	No. of contigs	GC content	Total CDSs	Completeness	Contamination
1	*Achromobacter veterisilvae* AB1	*Achromobacter*	*Achromobacter veterisilvae*	4	6 549 044	1	0.67	5991	100	0.98
2	*Ochrobactrum teleogrylli* AB1	*Ochrobactrum*	*Ochrobactrum_B teleogrylli*	1	5 064 753	3	0.57	4714	99.97	1.41
3	*Pseudomonas fluorescens* AB1	*Pseudomonas*	*Pseudomonas_E fluorescens_BH*	2	6 759 727	1	0.6	6053	100	0.15
4	*Stenotrophomonas* sp. AB1 (2024)	*Stenotrophomonas*	*Stenotrophomonas* spp.	1	3 926 254	2	0.66	3384	100	0.04
5	*Variovorax* sp. AB1 (2024)	*Variovorax*	*Variovorax* sp003019815	1	6 611 336	2	0.68	6041	99.99	0.37

The authors apologize for any inconvenience caused.

