# Supplementary material for: Characterizing a stable five-species microbial community for use in experimental evolution and ecology
Source: Microbiology (Reading). 2024 Sep 19;170(9):001489. doi: 10.1099/mic.0.001489 (PMC11412253; doi:10.1099/mic.0.001489)
Supplement: Uncited Supplementary Material 1. [file mic-170-01489-s001.pdf]

Supplementary Information for: Characterising a stable five-species microbial community for use in experimental evolution and ecology.

Author Affiliations

Meaghan Castledine[1], Joseph Pennycook[2], Arthur Newbury[1], Luke Lear[1], Zoltan Erdos[1], Rai Lewis[1], Suzanne Kay[1], Dirk Sanders[1], David Sünderhauf[1], Angus Buckling[1], Elze Hesse[1], & Daniel Padfield[1].

[1]Centre for Ecology and Conservation, College of Life and Environmental Sciences, University of Exeter, Penryn, Cornwall, TR10 9FE, U.K.

[2]University College Cork, Cork T12 K8AF

Corresponding author: Daniel Padfield (d.padfield@exeter.ac.uk)

Supplementary Methods and Results

Description of colony morphotypes of the five species on King's Medium (KB) agar

- *Achromobacter* sp. colonies are typically the smallest of the five species, being uniform in size and transparent when held up to the light.
- *Ochrobactrum* sp. colonies are similar to *Achromobacter* sp. but are typically larger and opaque when held up to the light. Colony size is typically uniform.
- *Pseudomonas* sp. colonies are typically the largest and are cream in colour which distinguishes them from *Ochrobactrum* sp. Colonies are opaque when held up to the light and are uniform in size.
- *Stenotrophomonas* sp. colonies are distinguished by their dense centre, followed by a lighter ring and dense outer-edge. Colonies are transparent when held up to the light. Colony size is variable within a culture.
- *Variovorax* sp. colonies are transparent colonies pale yellow in colour. Appearance is typified by a dense colony center followed by a thin mat surrounding the colony. Unlike *Stenotrophomonas* sp, the outer colony edge is not dense. Colony size is variable within a culture.

Analysing the community composition of the (dis)assembled communities

To measure the community composition of the 48 (dis)assembled communities, we sequenced the 16S rRNA gene. DNA was extracted by following the Qiagen's standard DNeasy Blood and Tissue kit. A conserved fragment from the hypervariable region of the 16S rRNA gene was targeted using N501F and N806R primers and amplicon sequencing was done using Illumina sequencing to create 2x250bp paired end reads.

We processed and analysed the sequence data in R using the packages *dada2* and *phyloseq*. Following the standard full stack workflow, we estimated error rates, inferred and merged sequences, constructed a sequence table, removed chimeric sequences and assigned taxonomy. Assembled amplicon sequence variants (ASVs) were assigned taxonomy using the RDP database. A phylogenetic tree was estimated using *fasttree*. This pipeline resulted in 41 ASVs across the 48 samples. To filter out rare ASVs, we filtered out ASVs that had a minimum total abundance of 200 across the whole dataset (total reads=36,365,589) or were present in only fewer than 5% samples. This left us with 18 ASVs.

To look at how prevalent and common our 5 species were across these replicates, we extracted the 16S sequences from each of their reference genomes and assigned taxonomy of

each of the 18 ASVs based purely on those sequences. We only allowed exact sequence matches, and each reference genome was only assigned to one ASV. We filtered each replicate to only include ASVs at a proportion >0.001 (0.1%), and then calculated the prevalence (presence/absence) and mean relative abundance for each ASV.

The species in the model community dominate, but Pseudomonas fluorescens AB1 strongly competes with another Pseudomonas.

Of the 5 species in the model community, 4 of them were present in every community, with *Pseudomonas* sp. only being present in 90% of the communities. A different *Pseudomonas* ASV (Pseudomonas 1) was present in every community. To look at how dominant the 5 species in our model community were in the (dis)assembled communities, we visualised the rank-abundance curves of each community (Figure S1a). The five species were consistently the most abundance ASVs in the communities (Figure S1a), but there was an ASV not present in the model community that was also regularly dominating the community. To investigate this further, we looked at the relative abundance of each ASV in each community. The relative abundance of species in the model community (Figure S1b) was consistently higher than those of ASVs not in the model community (Figure S1c). *Pseudomonas* sp. was regularly the most abundant species in the community (Figure S1b), but also had many replicates where it had a much lower abundance. A *Pseudomonas* ASV not in the model community (*Pseudomonas* 1) demonstrated a similar pattern (Figure S1c). To look at the relationship between Pseudomonad ASVs, we plotted their relative abundance across and within replicates (Figure S1d). *Pseudomonas* sp. and *Pseudomonas* 1 are negatively correlated, when one dominates the community, the other is either absent or at very low abundance. This indicates that they are strong competitors and functionally similar, but also that some stochastic process determines which one remains in the (dis)assembled community. Overall, these results demonstrate that the communities (dis)assembled in a deterministic way, and were generally dominated by 5 ASVs, with one of 2 Pseudomonads generally being the dominant community member.

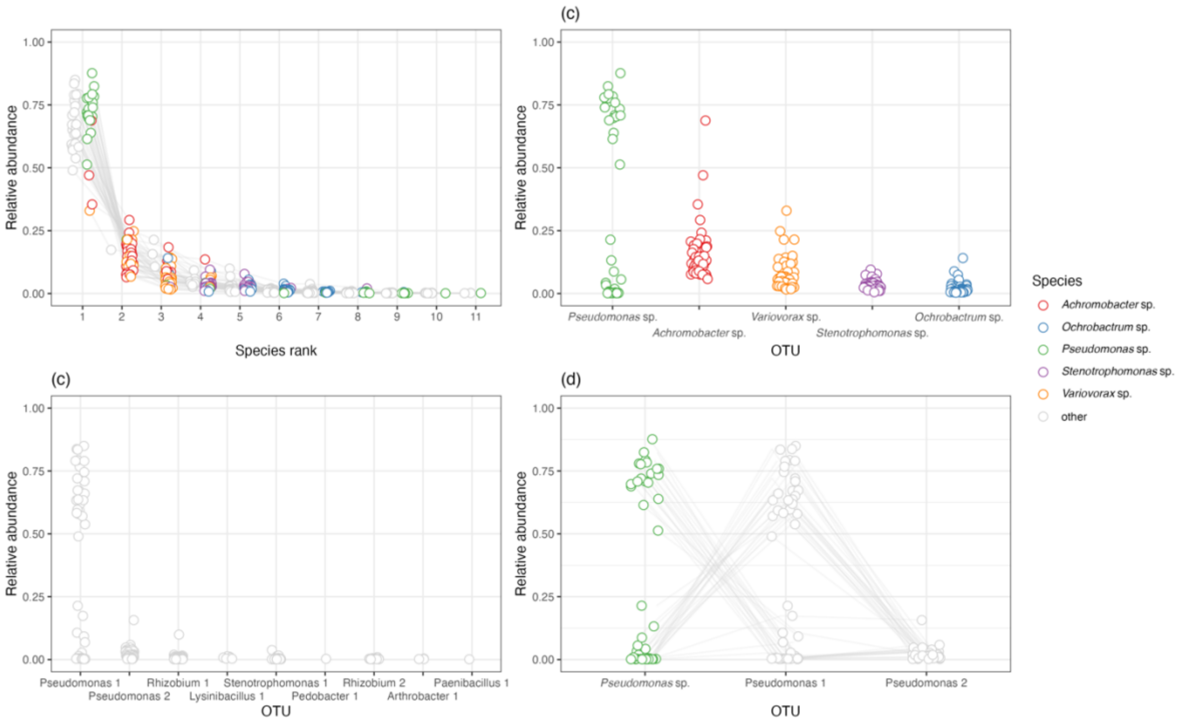

Figure S1. The composition of the 48 (dis)assembled communities is dominated by the 5 species in the model community. (a) Rank abundance curves of each of the 48 communities. Relative abundance of each ASV across the 48 communities for (b) species in the model community and (c) ASVs not in the model community. (d) Relative abundance of the *Pseudomonas* ASVs in each of the communities. The relative abundance of *Pseudomonas* sp. from the model community is negatively correlated with that of one of the other *Pseudomonas* sp., indicating that they strongly compete and are functionally similar. Points represent individual community replicates. Coloured points represent ASVs present in the five species model community, with grey points representing other ASVs. Grey lines are used to join species that come from the same replicate.

Table S1. The 46 bacterial strains used to establish the stable model community.

Code	Family	Genus
NMC6xB_4	Alcaligenaceae	Achromobacter
NMC5_19	Alcaligenaceae	Achromobacter
NMC1_18	Comamonadaceae	Acidovorax
NMC6_14	Microbacteriaceae	Agromyces
NMC2x_9	Micrococcaceae	Arthrobacter
NMC1_7	Micrococcaceae	Arthrobacter
NMC5_4	Bacillaceae	Bacillus
NMC5_14	Bacillaceae	Bacillus
NMC1B_24	Alcaligenaceae	Bordetella
NMC5xB_11	Alcaligenaceae	Bordetella
NMC5_15	Caulobacteraceae	Brevundimonas
NMC6x_4	Caulobacteraceae	Brevundimonas
NMC1B_19	Alcaligenaceae	Candidimonas
NMC6_15	Alcaligenaceae	Candidimonas
NMC2xB_19	Burkholderiaceae	Cupriavidus
NMC1_10	Burkholderiaceae	Cupriavidus
NMC1B_16	Hyphomicrobiaceae	Devosia
NMC5_2	Hyphomicrobiaceae	Devosia
NMC5x_2	Flavobacteriaceae	Flavobacterium
NMC5x_7	Flavobacteriaceae	Flavobacterium
NMC4_13	Planococcaceae	Lysinibacillus
NMC3_4	Planococcaceae	Lysinibacillus
NMC5B_21	Microbacteriaceae	Microbacterium
NMC5_10	Microbacteriaceae	Microbacterium
NMC4_4	Brucellaceae	Ochrobactrum
NMC6B_13	Cellulomonadaceae	Oerskovia
NMC2B_2	Cellulomonadaceae	Oerskovia
NMC4_22	Paenibacillaceae	Paenibacillus
NMC4x_9	Paenibacillaceae	Paenibacillus
NMC6xB_20	Rhodobacteraceae	Paracoccus
NMC5_5	Rhodobacteraceae	Paracoccus
NMC5_17	Sphingobacteriaceae	Pedobacter
NMC5_21	Alcaligenaceae	Pigmentiphaga
NMC5_3	Pseudomonadaceae	Pseudomonas
NMC6B_1	Pseudomonadaceae	Pseudomonas
NMC6B_4	Alcaligenaceae	Pusillimonas
NMC5_9	Alcaligenaceae	Pusillimonas
NMC4B_5	Rhizobiaceae	Rhizobium
NMC4B_1	Nocardiaceae	Rhodococcus
NMC4B_16	Nocardiaceae	Rhodococcus
NMC4B_3	Rhodocyclaceae	Shinella
NMC3xB_22	Staphylococcaceae	Staphylococcus
NMC5_6	Xanthomonodaceae	Stenotrophomonas
NMC5_23	Xanthomonodaceae	Stenotrophomonas
NMC1_21	Comamonadaceae	Variovorax
NMC6_9	Comamonadaceae	Variovorax

Table S2. Equations from calibration curves to estimate each species' CFU uL^{-1} from optical density (OD) readings.

species	equation
Achromobacter sp.	(11676.OD - 291).16667
Ochrobactrum sp.	(5548.OD - 67).16667
Pseudomonas sp.	(546.OD - 20).16667
Stenotrophomonas sp.	(56625.OD - 2246).16667
Variovorax sp.	(955.OD - 30).16667
